# Supplementary material for: Functional Resilience of the Neural Visual Recognition System Post-Pediatric Occipitotemporal Resection
Source: bioRxiv. 2024 May 8:2024.05.08.592792. Preprint. [Version 1] doi: 10.1101/2024.05.08.592792 (PMC11100714; doi:10.1101/2024.05.08.592792)
Supplement: Supplement 1 [file media-1.pdf]

Supplemental Information

Table S1: *Model Selection (Category Selectivity)*

Models			
#	Model		AIC
1	CS ~ group * hemi * stim + age + (1|ID)		326.66
2	CS ~ group * hemi + group * stim + hemi * stim + age + (1|ID)		322.14
3	CS ~ group * hemi + group * stim + age + (1|ID)		320.79
4	CS ~ group * hemi + hemi * stim + age + (1|ID)		316.95
5	CS ~ group * stim + hemi * stim + age + (1|ID)		320.84
6	CS ~ hemi * stim + group + age + (1|ID)		315.73
7	CS ~ group * hemi + stim + age + (1|ID)		315.11
8	CS ~ group * stim + hemi + age + (1|ID)		319.59
9	**CS ~ group + stim + hemi + age + (1|ID)**		313.98
10	CS ~ group + hemi + age + (1|ID)		316.83
11	CS ~ group + stim + age + (1|ID)		311.98
LRTs			
# vs. #	χ^2	Df	*p*
1 vs. 2	3.48	4	0.48
2 vs. 3	2.65	2	0.27
2 vs. 4	2.81	4	0.59
2 vs. 5	2.70	2	0.26
3 vs. 4	0.16	2	0.92
4 vs. 5	0.11	2	0.95
3 vs. 7	2.32	4	0.68
3 vs. 8	2.80	2	0.25
4 vs. 6	2.78	2	0.25
4 vs. 7	2.16	2	0.34
5 vs. 6	2.90	4	0.58
5 vs. 8	2.75	2	0.25
6 vs. 8	0.14	2	0.93
7 vs. 8	0	2	~ 1
6 vs. 9	2.25	2	0.33
7 vs. 9	2.87	2	0.24
8 vs. 9	2.39	4	0.66
9 vs. 10	6.85	2	**0.03**
9 vs. 11	< 0.01	1	0.97

Selected model and significant *p*-value are bolded. LRT = likelihood ratio test. AIC = Akaike information criterion. Df = degrees of freedom. CS = category selectivity amplitude. hemi = hemisphere. stim = stimulus category.

Table S2: *Post Hoc Comparisons (Category Selectivity ~ Stimulus Category)*

Comparison	*z*	*p*
Faces - Objects	0.51	0.61
Faces - Words	2.55	**0.03**
Objects - Words	2.07	0.06

Significant *p*-value is bolded.

Table S3: *Crawford Statistics (Category Selectivity, Repetition Suppression, Behavior)*

ID	Stimulus Category	*p* (comparison	*p* (comparison	*p* (comparison	*p* (comparison	*p* (comparison

		to TD LH on CS)	to TD RH on CS)	to TD LH on RS)	to TD RH on RS)	to TD on behavior)
Preserved LH OTC Patients						
sub-004	Faces	0.84	0.84	0.86	0.85	0.74
	Objects	0.82	0.84	0.83	0.80	0.75
	Words	-	-	-	-	0.84
sub-077	Faces	0.84	0.84	0.82	0.85	0.63
	Objects	0.82	0.84	0.20	0.80	0.30
	Words	0.79	0.83	0.85	0.79	0.49
sub-079	Faces	0.84	0.84	0.86	0.85	0.74
	Objects	0.82	0.84	0.83	0.80	0.62
	Words	0.79	0.83	0.85	0.79	0.84
sub-089	Faces	0.84	0.84	0.52	0.70	**0.01**
	Objects	0.82	0.84	0.83	0.80	0.27
	Words	0.83	0.83	0.85	0.79	0.84
sub-091	Faces	0.84	0.84	0.53	0.85	0.63
	Objects	0.82	0.84	0.83	0.80	0.15
	Words	0.83	0.83	0.85	0.79	0.84
Preserved RH OTC Patients						
sub-066	Faces	0.84	0.84	0.42	0.70	**0.03**
	Objects	0.82	0.84	0.86	0.80	-
	Words	0.83	0.83	0.85	0.79	0.49
sub-069	Faces	-	-	-	-	**0.01**
	Objects	0.82	0.84	0.83	0.80	-
	Words	0.63	0.83	0.85	0.79	**0.01**
sub-090	Faces	0.84	0.84	0.86	0.85	0.74
	Objects	0.82	0.84	0.83	0.80	0.20
	Words	0.79	0.83	0.85	0.79	0.84
sub-092	Faces	0.84	0.84	0.86	0.85	0.07
	Objects	0.82	0.84	0.83	0.80	0.41
	Words	0.63	0.83	0.85	0.79	0.84
Preserved LH Control Patients						
sub-072	Faces	0.84	0.84	0.86	0.85	0.74
	Objects	0.82	0.84	0.83	0.80	0.20
	Words	0.63	0.83	0.85	0.79	0.84
sub-073	Faces	0.84	0.84	0.86	0.85	0.74
	Objects	0.82	0.84	0.83	0.80	0.75
	Words	0.83	0.83	0.85	0.79	0.84

sub-075	Faces	0.84	0.84	0.53	0.70	**0.01**
	Objects	0.82	0.84	0.58	0.80	0.20
	Words	-	-	-	-	-
sub-080	Faces	0.84	0.84	0.86	0.85	0.74
	Objects	0.82	0.84	0.83	0.80	0.41
	Words	-	-	-	-	0.84
sub-086	Faces	0.84	0.84	0.86	0.85	0.84
	Objects	0.82	0.84	0.83	0.80	0.71
	Words	0.63	0.83	0.69	0.32	0.84
Preserved RH Control Patients						
sub-007	Faces	0.84	0.84	0.86	0.85	0.74
	Objects	0.82	0.84	0.83	0.80	0.75
	Words	-	-	-	-	0.84
sub-045	Faces	0.84	0.84	0.86	0.85	0.63
	Objects	0.82	0.84	0.83	0.80	0.75
	Words	-	-	-	-	0.84
sub-070	Faces	0.84	0.84	0.53	0.85	0.74
	Objects	0.82	0.84	0.83	0.80	0.58
	Words	0.63	0.83	0.85	0.79	0.84
sub-076	Faces	0.84	0.84	0.42	0.70	0.74
	Objects	-	-	-	-	0.58
	Words	-	-	-	-	0.84
sub-078	Faces	0.84	0.84	**0.01**	0.11	0.74
	Objects	0.82	0.84	0.83	0.80	0.51
	Words	0.83	0.83	0.85	0.79	0.84
sub-081	Faces	0.84	0.84	0.86	0.85	0.63
	Objects	0.82	0.84	0.83	0.80	0.58
	Words	0.83	0.83	0.85	0.79	0.84
sub-082	Faces	0.84	0.84	0.86	0.85	0.74
	Objects	0.82	0.84	0.83	0.80	0.58
	Words	0.83	0.83	0.85	0.79	0.84

Significant *p*-values are bolded. TD = typically developing controls. LH = left hemisphere. RH = right hemisphere. CS = category selectivity amplitude. RS = repetition suppression magnitude.

Table S4: *Model Selection (Responsivity to Adaptation Experiment*)

Models		
#	Model	AIC
1	resp ~ group * cond * hemi * stim + age + (1|ID)	445.17
2	**resp ~ group * cond * hemi + group * cond * stim + group * hemi * stim + cond * hemi * stim + age + (1|ID)**	430.30
3	resp ~ group * cond * stim + group * hemi * stim + cond * hemi * stim + age + (1|ID)	423.17

4	resp ~ group * cond * hemi + group * hemi * stim + cond * hemi * stim + age + (1|ID)	416.86
5	resp ~ group * cond * hemi + group * cond * stim + cond * hemi * stim + age + (1|ID)	427.31
6	resp~ group * cond * hemi + group * cond * stim + group * hemi * stim + age + (1|ID)	423.59
7	resp ~ group * cond * hemi + group * cond * stim + age + (1|ID)	428.72
8	resp ~ group * cond * hemi + group * hemi * stim + age + (1|ID)	403.18
9	resp ~ group * cond * hemi + cond * hemi * stim + age + (1|ID)	416.86
10	resp ~ group * cond * stim + group * hemi * stim + age + (1|ID)	412.54
11	resp ~ group * cond * stim + cond * hemi * stim + age + (1|ID)	420.72
12	resp ~ group * hemi * stim + cond * hemi * stim + age + (1|ID)	401.93

LRTs

# vs. #	χ^2	Df	*p*
1 vs. 2	1.13	8	~ 1
2 vs. 3	0.87	4	0.93
2 vs. 4	2.56	8	0.96
2 vs. 5	5.01	4	0.29
2 vs. 6	1.29	4	0.86
3 vs. 4	1.69	4	0.79
4 vs. 5	0	4	~ 1
4 vs. 6	1.27	4	0.87
3 vs. 10	1.37	6	0.97
3 vs. 11	9.55	6	0.14
3 vs. 12	2.76	12	~ 1
4 vs. 8	2.32	8	0.97
4 vs. 9	15.41	8	0.05
4 vs. 12	1.07	8	~ 1
5 vs. 7	13.41	6	**0.04**
5 vs. 9	12.97	12	0.37
5 vs. 11	5.41	6	0.49
6 vs. 7	17.13	6	**0.01**
6 vs. 8	3.59	12	0.99
6 vs. 10	0.94	6	0.99

Selected model and significant *p*-value are bolded. Note that since models 5 and 6 both better fit the data than model 7 with fewer parameters, and since model 2 is the model with the fewest parameters that nonetheless contains all the parameters in models 5 and 6, model 2 was selected. LRT = likelihood ratio test. AIC = Akaike information criterion. Df = degrees of freedom. resp = responsivity. cond = condition (same, alternating, different). hemi = hemisphere. stim = stimulus category.

Table S5: *Post Hoc Comparisons (Responsivity ~ Hemisphere x Stimulus Category)*

Comparison	*z*	*p*
LH Faces - RH Faces	0.14	0.99
LH Faces - LH Objects	3.71	**< 0.001**
LH Faces - LH Words	-4.16	**< 0.001**
RH Faces - RH Objects	4.92	**< 0.001**
RH Faces - RH Words	0.02	0.99
LH Objects - RH Objects	1.15	0.32
LH Objects - LH Words	-7.42	**< 0.001**
RH Objects - RH Words	-3.81	**< 0.001**
LH Words - RH Words	2.89	**0.01**

Significant *p*-values are bolded. LH = left hemisphere. RH = right hemisphere.

Table S6: *Post Hoc Comparisons (Responsivity ~ Group x Stimulus Category)*

Stimulus Category	Comparison	*z*	*p*	*p* (permutation testing)
Faces	OTC - TD	-0.13	0.96	0.95
	OTC - CP	-0.08	0.96	0.95
	TD - CP	0.05	0.96	0.95
Objects	OTC - TD	-0.59	0.96	0.95
	OTC - CP	0.36	0.96	0.95
	TD - CP	1.09	0.83	0.79
Words	OTC - TD	-0.54	0.96	0.95
	OTC - CP	-1.96	0.30	0.79
	TD - CP	-1.84	0.30	0.79

OTC = occipitotemporal cortex patients. TD = typically developing controls. CP = control patients.

Table S7: *Crawford Statistics (Responsivity to Adaptation Experiment)*

ID	Stimulus Category	Condition	*p* (comparison to TD LH)	*p* (comparison to TD RH)
Preserved LH OTC Patients				
sub-004	Faces	Same	0.91	0.90
		Alt	0.91	0.90
		Diff	0.91	0.90
	Objects	Same	0.88	0.91
		Alt	0.88	0.91
		Diff	0.88	0.91
	Words	Same	-	-
		Alt	-	-
		Diff	-	-
sub-077	Faces	Same	0.89	0.91
		Alt	0.89	0.91
		Diff	0.65	0.90
	Objects	Same	0.88	0.91
		Alt	0.75	0.91
		Diff	**0.01**	0.91
	Words	Same	0.88	0.89
		Alt	0.88	0.89
		Diff	0.85	0.89
sub-079	Faces	Same	0.91	0.91
		Alt	0.91	0.90
		Diff	0.91	0.92
	Objects	Same	0.88	0.91
		Alt	0.91	0.91
		Diff	0.88	0.91
	Words	Same	0.88	0.89

		Alt	0.88	0.89
		Diff	0.88	0.89
sub-089	Faces	Same	0.91	0.92
		Alt	0.91	0.92
		Diff	0.91	0.92
	Objects	Same	0.88	0.91
		Alt	0.88	0.91
		Diff	0.88	0.91
	Words	Same	0.85	0.89
		Alt	0.88	0.89
		Diff	0.88	0.89
sub-091	Faces	Same	0.91	0.91
		Alt	0.91	0.91
		Diff	0.91	0.91
	Objects	Same	0.88	0.91
		Alt	0.88	0.91
		Diff	0.88	0.91
	Words	Same	0.88	0.89
		Alt	0.85	0.89
		Diff	0.85	0.89
Preserved RH OTC Patients				
sub-066	Faces	Same	0.87	0.90
		Alt	0.87	0.90
		Diff	0.87	0.90
	Objects	Same	0.88	0.91
		Alt	0.88	0.91
		Diff	0.9	0.91
	Words	Same	0.81	0.89
		Alt	0.88	0.89
		Diff	0.85	0.89
sub-069	Faces	Same	-	-
		Alt	-	-
		Diff	-	-
	Objects	Same	0.91	0.91
		Alt	0.91	0.91
		Diff	0.90	0.91
	Words	Same	0.85	0.89
		Alt	0.85	0.89
		Diff	0.88	0.89

sub-090	Faces	Same	0.87	0.90
		Alt	0.91	0.90
		Diff	0.91	0.90
	Objects	Same	0.88	0.91
		Alt	0.88	0.91
		Diff	0.90	0.91
	Words	Same	0.85	0.89
		Alt	0.85	0.89
		Diff	0.85	0.89
sub-092	Faces	Same	0.87	0.90
		Alt	0.65	0.90
		Diff	0.65	0.90
	Objects	Same	0.65	0.91
		Alt	0.88	0.91
		Diff	0.88	0.91
	Words	Same	0.88	0.89
		Alt	0.88	0.89
		Diff	0.91	0.89
Preserved LH Control Patients				
sub-072	Faces	Same	0.91	0.92
		Alt	0.91	0.92
		Diff	0.89	0.90
	Objects	Same	0.88	0.91
		Alt	0.88	0.91
		Diff	0.88	0.91
	Words	Same	0.85	0.89
		Alt	0.85	0.89
		Diff	0.81	0.89
sub-073	Faces	Same	0.84	0.90
		Alt	0.81	0.90
		Diff	0.65	0.90
	Objects	Same	0.91	0.91
		Alt	0.90	0.91
		Diff	0.88	0.91
	Words	Same	0.88	0.89
		Alt	0.88	0.89
		Diff	0.85	0.89
sub-075	Faces	Same	0.91	0.90
		Alt	0.91	0.90

		Diff	0.91	0.90
	Objects	Same	0.88	0.91
		Alt	0.88	0.91
		Diff	0.88	0.91
	Words	Same	-	-
		Alt	-	-
		Diff	-	-
sub-080	Faces	Same	0.91	0.92
		Alt	0.91	0.91
		Diff	0.87	0.90
	Objects	Same	0.88	0.91
		Alt	0.88	0.91
		Diff	0.88	0.91
	Words	Same	-	-
		Alt	-	-
		Diff	-	-
sub-086	Faces	Same	0.87	0.90
		Alt	0.91	0.90
		Diff	0.91	0.90
	Objects	Same	0.88	0.91
		Alt	0.88	0.91
		Diff	0.88	0.91
	Words	Same	0.88	0.89
		Alt	0.85	0.89
		Diff	0.81	0.89
Preserved RH Control Patients				
sub-007	Faces	Same	0.91	0.90
		Alt	0.91	0.90
		Diff	0.91	0.91
	Objects	Same	0.88	0.91
		Alt	0.91	0.91
		Diff	0.88	0.91
	Words	Same	-	-
		Alt	-	-
		Diff	-	-
sub-045	Faces	Same	0.91	0.90
		Alt	0.91	0.90
		Diff	0.91	0.91
	Objects	Same	0.90	0.91

		Alt	0.90	0.91
		Diff	0.90	0.91
	Words	Same	-	-
		Alt	-	-
		Diff	-	-
sub-070	Faces	Same	0.91	0.90
		Alt	0.91	0.90
		Diff	0.91	0.92
	Objects	Same	0.88	0.91
		Alt	0.88	0.91
		Diff	0.91	0.91
	Words	Same	0.88	0.89
		Alt	0.85	0.89
		Diff	0.88	0.89
sub-076	Faces	Same	0.89	0.90
		Alt	0.89	0.90
		Diff	0.89	0.90
	Objects	Same	-	-
		Alt	-	-
		Diff	-	-
	Words	Same	-	-
		Alt	-	-
		Diff	-	-
sub-078	Faces	Same	0.91	0.90
		Alt	0.65	0.90
		Diff	0.06	0.50
	Objects	Same	0.88	0.91
		Alt	0.91	0.91
		Diff	0.88	0.91
	Words	Same	0.88	0.89
		Alt	0.88	0.89
		Diff	0.85	0.89
sub-081	Faces	Same	0.91	0.90
		Alt	0.91	0.90
		Diff	0.91	0.91
	Objects	Same	0.88	0.91
		Alt	0.88	0.91
		Diff	0.89	0.91
	Words	Same	0.85	0.89

		Alt	0.85	0.89
		Diff	0.81	0.89
sub-082	Faces	Same	0.91	0.91
		Alt	0.91	0.91
		Diff	0.91	0.92
	Objects	Same	0.91	0.91
		Alt	0.9	0.91
		Diff	0.88	0.91
	Words	Same	0.85	0.89
		Alt	0.85	0.89
		Diff	0.85	0.89

Significant *p*-value is bolded. TD = typically developing controls. LH = left hemisphere. RH = right hemisphere. CS = category selectivity amplitude. RS = repetition suppression magnitude.

Table S8: *Model Selection (Repetition Suppression)*

Models			
#	Model		AIC
1	RS ~ group * hemi * stim + age + (1|ID)		-70.69
2	**RS ~ group * hemi + group * stim + hemi * stim + age + (1|ID)**		-75.06
3	RS ~ group * hemi + group * stim + age + (1|ID)		-70.27
4	RS ~ group * hemi + hemi * stim + age + (1|ID)		-74.82
5	RS ~ group * stim + hemi * stim + age + (1|ID)		-76.61
**LRTs**			
# vs. #	χ^2	Df	*p*
1 vs. 2	3.63	4	0.46
2 vs. 3	8.79	2	**0.01**
2 vs. 4	8.24	4	0.08
2 vs. 5	2.45	2	0.29

Selected model and significant *p*-value are bolded. LRT = likelihood ratio test. AIC = Akaike information criterion. Df = degrees of freedom. RS = repetition suppression magnitude. hemi = hemisphere. stim = stimulus category.

Table S9: *Post Hoc Comparisons (RS Magnitude ~ Hemisphere x Stimulus Category)*

Comparison	*z*	*p*
LH Faces - RH Faces	-0.03	0.98
LH Faces - LH Objects	-0.76	0.67
LH Faces - LH Words	-3.84	**< 0.01**
RH Faces - RH Objects	-1.29	0.44
RH Faces - RH Words	-0.74	0.69
LH Objects - RH Objects	-0.50	0.73
LH Objects - LH Words	-3.15	**0.01**
RH Objects - RH Words	0.45	0.73
LH Words - RH Words	2.51	**0.04**

Significant *p*-values are bolded. RS = repetition suppression. LH = left hemisphere. RH = right hemisphere.

Table S10: *Model Selection (RSA Model Fitting)*

Models		
#	Model	AIC
1	model fit ~ group * hemi * stim + age + (1|ID)	-96.49

2	model fit ~ group * hemi + group * stim + hemi * stim + age + (1|ID)	-98.87
3	model fit ~ group * hemi + group * stim + age + (1|ID)	-100.99
4	model fit ~ group * hemi + hemi * stim + age + (1|ID)	-106.76
5	model fit ~ group * stim + hemi * stim + age + (1|ID)	-102.02
6	model fit ~ hemi * stim + group + age + (1|ID)	-109.91
7	model fit ~ group * hemi + stim + age + (1|ID)	-108.91
8	model fit ~ group * stim + hemi + age + (1|ID)	-104.14
9	**model fit ~ group + stim + hemi + age + (1|ID)**	-112.06
10	model fit ~ group + hemi + age + (1|ID)	166.69
11	model fit ~ group + stim + age + (1|ID)	-110.41

LRTs

# vs. #	χ^2	Df	*p*
1 vs. 2	5.63	4	0.23
2 vs. 3	1.88	2	0.39
2 vs. 4	0.11	4	~ 1
2 vs. 5	0.85	2	0.65
3 vs. 4	< 0.001	2	~ 1
4 vs. 5	< 0.001	2	~ 1
3 vs. 7	0.08	4	~ 1
3 vs. 8	0.85	2	0.65
4 vs. 6	0.85	2	0.65
4 vs. 7	1.85	2	0.40
5 vs. 6	0.11	4	~ 1
5 vs. 8	1.88	2	0.39
6 vs. 8	< 0.001	2	~ 1
7 vs. 8	< 0.001	2	~ 1
6 vs. 9	1.85	2	0.40
7 vs. 9	0.85	2	0.65
8 vs. 9	0.08	4	~ 1
9 vs. 10	282.75	2	**< 0.001**
9 vs. 11	3.65	1	0.06

Selected model and significant *p*-value are bolded. RSA = representational similarity analysis. LRT = likelihood ratio test. AIC = Akaike information criterion. Df = degrees of freedom. hemi = hemisphere. stim = stimulus category.

Table S11: *Crawford Statistics (RSA Model Fitting)*

ID	Model	*p* (comparison to TD LH)	*p* (comparison to TD RH)
Preserved LH OTC Patients			
sub-004	CS	0.86	0.68
	RS	0.85	0.74
	CS-RS	0.87	0.71
sub-077	CS	0.86	0.68
	RS	0.85	0.79
	CS-RS	0.87	0.71
sub-079	CS	0.86	0.78
	RS	0.85	0.74
	CS-RS	0.87	0.83
sub-089	CS	0.86	0.68
	RS	0.25	0.71

	CS-RS	0.87	0.71
sub-091	CS	0.86	0.78
	RS	0.85	0.74
	CS-RS	0.87	0.83
Preserved RH OTC Patients			
sub-066	CS	0.86	0.68
	RS	0.2	0.23
	CS-RS	0.87	0.71
sub-069	CS	0.86	0.78
	RS	0.85	0.74
	CS-RS	0.87	0.83
sub-090	CS	0.86	0.85
	RS	0.61	0.74
	CS-RS	0.87	0.85
sub-092	CS	0.86	0.85
	RS	0.2	0.39
	CS-RS	0.87	0.85
Preserved LH Control Patients			
sub-072	CS	0.86	0.85
	RS	0.85	0.79
	CS-RS	0.87	0.83
sub-073	CS	0.86	0.68
	RS	0.85	0.74
	CS-RS	0.87	0.71
sub-075	CS	0.86	0.68
	RS	0.31	0.74
	CS-RS	0.87	0.71
sub-080	CS	0.86	0.68
	RS	**0.01**	0.12
	CS-RS	0.87	0.71
sub-086	CS	0.86	0.68
	RS	0.85	0.74
	CS-RS	0.87	0.71
Preserved RH Control Patients			
sub-007	CS	0.86	0.68
	RS	0.85	0.82
	CS-RS	0.87	0.71
sub-045	CS	0.86	0.78

	RS	0.85	0.74
	CS-RS	0.87	0.83
sub-070	CS	0.86	0.68
	RS	0.20	0.62
	CS-RS	0.87	0.71
sub-076	CS	0.86	0.68
	RS	0.21	0.23
	CS-RS	0.87	0.71
sub-078	CS	0.86	0.78
	RS	0.61	0.71
	CS-RS	0.87	0.83
sub-081	CS	0.86	0.78
	RS	0.85	0.74
	CS-RS	0.87	0.83
sub-082	CS	0.86	0.78
	RS	0.85	0.74
	CS-RS	0.87	0.83

Significant *p*-value is bolded. TD = typically developing controls. LH = left hemisphere. RH = right hemisphere. CS = category selectivity. RS = repetition suppression.

Table S12: *Model Selection (Behavior)*

Models			
#	Model		AIC
1	**accuracy ~ group * stim + age + (1|ID)**		-181.60
2	accuracy ~ group + stim + age + (1|ID)		-174.36
**LRTs**			
# vs. #	χ^2	Df	*p*
1 vs. 2	15.25	4	**< 0.01**

Selected model and significant *p*-value are bolded. LRT = likelihood ratio test. AIC = Akaike information criterion. Df = degrees of freedom. stim = stimulus category.

Table S13: *Post Hoc Comparisons (Behavioral Accuracy ~ Group x Stimulus Category)*

Stimulus Category	Comparison	*z*	*p*	*p* (permutation testing)
Faces	OTC - TD	-4.06	**< 0.001**	**< 0.001**
	OTC - CP	-3.67	**< 0.01**	**< 0.001**
	TD - CP	0.10	0.92	0.93
Objects	OTC - TD	-5.37	**< 0.001**	**< 0.001**
	OTC - CP	-3.45	**< 0.01**	**0.03**
	TD - CP	2.02	0.08	0.27
Words	OTC - TD	-0.90	0.55	0.27
	OTC - CP	-0.70	0.62	0.31
	TD - CP	0.14	0.92	0.93

Significant *p*-values are bolded. OTC = occipitotemporal cortex patients. TD = typically developing controls. CP = control patients.

Table S14: *Model Selection (Behavior - Patient Data Only)*

Models		
#	Model	AIC
1	accuracy ~ group * hemi * stim + age + (1|ID)	-79.57
2	**accuracy ~ group * hemi + group * stim + hemi * stim + age + (1|ID)**	-80.79
3	accuracy ~ group * hemi + group * stim + age + (1|ID)	-84.58
4	accuracy ~ group * hemi + hemi * stim + age + (1|ID)	-78.74
5	accuracy ~ group * stim + hemi * stim + age + (1|ID)	-78.77

LRTs			
# vs. #	χ^2	Df	*p*
1 vs. 2	2.78	2	0.25
2 vs. 3	4.21	4	0.38
2 vs. 4	6.05	2	**0.05**
2 vs. 5	4.03	1	**0.05**

Selected model and significant *p*-value are bolded. LRT = likelihood ratio test. AIC = Akaike information criterion. Df = degrees of freedom. hemi = hemisphere. stim = stimulus category.

Table S15: *Post Hoc Comparisons (Behavioral Accuracy ~ Group x Hemisphere) (Patient Data Only)*

Comparison	*z*	*p*
CP LH - OTC LH	0.80	0.42
CP LH - OTC RH	-1.58	0.20
OTC LH - OTC RH	1.44	0.20
CP RH - OTC RH	3.83	**< 0.01**

Significant *p*-value is bolded. OTC = occipitotemporal cortex patients. CP = control patients. LH = left hemisphere. RH = right hemisphere.

Table S16: *Karolinska Directed Emotional Faces[1] Stimulus IDs*

AM02NES
AM03NES
AM05NES
AM06NES
AM07NES
AM10NES
AM11NES
AM13NES
AM17NES
AM18NES
AM21NES
AM23NES
AM25NES
AM26NES
AM27NES
AM31NES
AM32NES
AM35NES

Table S17: *R Packages*

broom[2]
car[3]

lme4[4]
nnet[5]
pracma[6]
psych[7]
singcar[8]
strex[9]
tidyverse[10]

References

1. Lundqvist, D., Flykt, A. & Öhman, A. *The Karolinska Directed Emotional Faces - KDEF*. (CD ROM from Department of Clinical Neuroscience, Psychology section, Karolinska Institutet, ISBN 91-630-7164-9, 1998).
2. Robinson, D. broom: An R Package for Converting Statistical Analysis Objects Into Tidy Data Frames. *arXiv* (2014).
3. Companion to Applied Regression [R package car version 3.0-12]. (2021). at <https://cran.r-project.org/web/packages/car/index.html>
4. Bates, D., Mächler, M., Bolker, B. & Walker, S. Fitting linear mixed-effects models using lme4. *J. Stat. Softw.* **67,** 1–48 (2015).
5. Venables, W. N. & Ripley, B. D. *Modern Applied Statistics with S (Statistics and Computing)*. 510 (Springer, 2002).
6. Borchers, H. W. *pracma: Practical Numerical Math Functions*. (2021).
7. Revelle, W. *psych: Procedures for Personality and Psychological Research*. (Northwestern Unviersity, 2021).
8. Rittmo, J. & McIntosh, R. *singcar: Comparing Single Cases to Small Samples*. (2021).
9. Nolan, R. *strex: Extra String Manipulation Functions*. (2021).
10. Wickham, H. *et al.* Welcome to the tidyverse. *JOSS* **4,** 1686 (2019).
